# Effect of opaque porcelain on color and aesthetic outcomes of titanium-abutment implant restorations

**DOI:** 10.1371/journal.pone.0330788

**Published:** 2025-09-12

**Authors:** Xiaodong Sun, Long Li, Hao Zhang, Hao Feng, Xingyu Liu, Yang Qiu, Cai Wen

**Affiliations:** 1 Department of Prosthodontics, The Affiliated Stomatology Hospital, Southwest Medical University, Luzhou, China; 2 Department of Oral Implantology, The Affiliated Stomatology Hospital, Southwest Medical University, Luzhou, China; 3 Luzhou Key Laboratory of Oral & Maxillofacial Reconstruction and Regeneration, Southwest Medical University, Luzhou, China; 4 Institute of Stomatology, Southwest Medical University, Luzhou, Sichuan, China; 5 Department of Oral and Maxillofacial Surgery, The Affiliated Stomatology Hospital, Southwest Medical University, Luzhou, China; 6 Department of Endodontics, The Affiliated Stomatology Hospital, Southwest Medical University, Luzhou, China; International Medical University, MALAYSIA

## Abstract

**Objective:**

This study evaluated the effects of opaque porcelain (OP) on color parameters and aesthetic outcomes of all-ceramic implant restorations with varying abutment types and restoration thicknesses.

**Methods:**

Four types of zirconia discs were fabricated:(A) 1.5 mm zirconia; (B) 1.4 mm zirconia + 0.1 mm OP; (C) 2.0 mm zirconia; (D) 1.9 mm zirconia + 0.1 mm OP. These discs were bonded to titanium (t) or all-ceramic (c) backgrounds to form At, Ac, Bt, Bc, Ct, Cc, Dt, and Dc specimens,to simulate the aesthetic results of implant restorations with or without OP on titanium or all-ceramic abutments. The assembled specimens were placed at the center of a dark backboard in a specialized constructed photography platform, and a professional camera was used to acquire images of the specimens. Eight regions of interest on the image were selected, and color parameters in the form of L*, a*, and b* values and variations determined via the CIELAB color measurement system were analyzed using Adobe Photoshop software.Color differences (ΔE) between samples were quantified. Statistical comparisons of color parameters were conducted using the Kruskal-Wallis H test and Welch’s *t*-test.

**Results:**

There were significant differences in L*, a*, and b* between all-ceramic and titanium backgrounds in all types of zirconia specimens (*p* < 0.001). The application of OP influenced the brightness and chroma of zirconia restorations on titanium backgrounds. ΔE values between restorations with metal substrate and OP, and those with all-ceramic substrate but without OP, (△E (Ac/Bt)= 1.99 ± 0.86; △E(Cc/Dt)= 1.31 ± 0.83) indicated that OP effectively mitigated the color compromise caused by metallic implant abutments. The greater the thickness of the restoration, the smaller the color difference caused by the titanium background.

**Conclusion:**

This study revealed that titanium abutments can affect the color of implant-supported restorations. The application of OP on restorations reduces the titanium abutment’s color impact, and this effect improves with increased zirconia thickness.

## 1. Introduction

The principal objective of implant restoration is to restore both tooth function and aesthetics. With the improved predictability of implant treatment, the acceptance of oral implantology has markedly increased [1]. Nevertheless, achieving natural aesthetics in implant restorations is a critical challenge, particularly when using titanium abutments that can compromise appearance.

Currently, whether in the field of implant or traditional tooth restorations, all-ceramic materials are predominantly used [2,3]. Because implant-supported prostheses have metal abutments, their gray metallic color may be visible through the mucosa and under prosthetic crowns, potentially compromising the final aesthetics when using all-ceramic restorations [4]. Achieving color and appearance consistency between implant restorations and natural teeth remains a significant challenge in oral implantology.

Implant abutments are used as connecting components between the restoration and implant body,and the mainstream abutment materials used in clinical practice are metallic titanium or all-ceramic zirconia [5]. Ceramic abutments have limited clinical applications owing to their drawbacks, such as high fragility and susceptibility to fracture [6]. Titanium abutments, made of pure titanium or titanium alloys, possess favorable mechanical properties and biocompatibility, along with low processing difficulty and manufacturing costs, making them a common choice for clinical implant abutments.

The use of zirconia as the prevailing all-ceramic material for dental crown restoration is common, and zirconia has the advantages of biocompatibility, high mechanical strength, high translucency, and stable coloration [7–10]. Nevertheless, all-ceramic restorations present certain limitations: owing to their good light transmission properties and relatively weak color-masking capacity, the metallic color of the titanium abutment underneath the restoration cannot be entirely concealed, possibly presenting a grayish-black metallic color through the restoration [11].

In traditional porcelain-fused-to-metal (PFM) crown production, the inner metal base can contribute to metallic color, and opaque porcelain (OP) is employed in dental laboratories [12]. The metal color transmission problem caused by the implant metal abutment is similar to that observed in the PFM crown. We believe that OP may also partially address this problem; however, rigorous research on the masking effect of OP on the metallic color of the abutment in implant restoration is lacking.

To optimize the aesthetic outcome of implant restoration, we used the CIELAB (1976) color system to quantify and compare its colors. The CIELAB system is an international standard color-representation method proposed by the International Commission on Illumination (CIE) in 1976 [13,14]. This system describes color in three dimensions: L* represents brightness, a* represents the change from green to red, and b* represents the change from blue to yellow. This three-dimensional representation makes it possible to precisely measure and compare the differences between colors.

In this study, we aimed to explore the effect of the application of OP on the color parameters (L*, a*, b*) and color difference (ΔE) of all-ceramic restoration, evaluate its effect on masking metallic color in implant prostheses, and compare the effects of OP with different prosthesis thicknesses. The null hypothesis (Tₒ) of this study is that OP can rescue the color change of all-ceramic zirconia implant restoration caused by the metal abutment.

## 2. Methods

In this study, titanium and all-ceramic backgrounds were used to simulate the titanium and all-ceramic implant abutments, respectively, and the upper zirconia disc was used to simulate zirconia implant restoration. A professional camera was employed to capture images of these restorations in diverse conditions using a standard photography procedure. Regions of interest (ROIs) were selected on the captured images, and their color parameters and variations were analyzed using image analysis software.As this study did not involve any research with human participants or animals, ethics approval and formal consent were not required.

**Fig 1:**
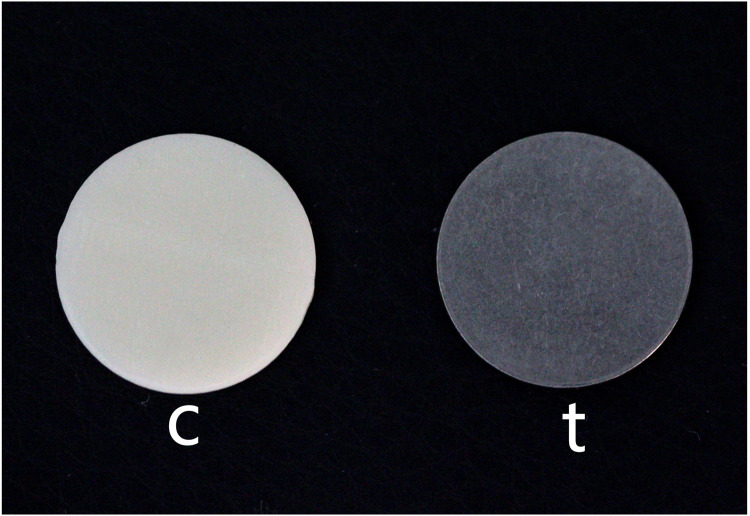
Titanium (t) and all-ceramic discs (c) were used to simulate titanium and all-ceramic abutment.

**Fig 2:**
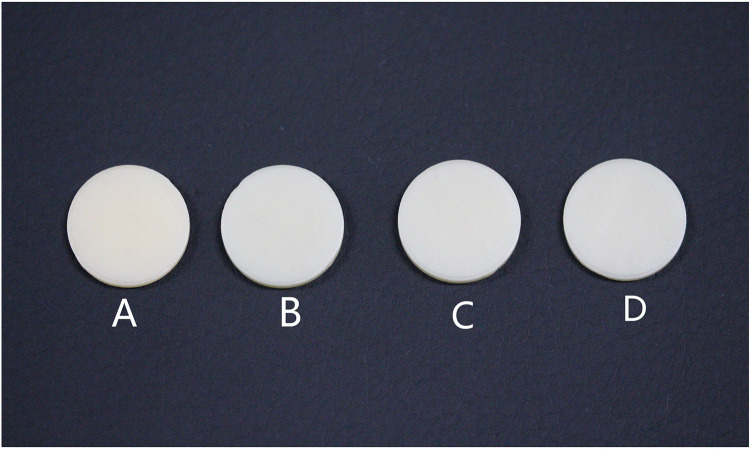
Zirconia discs with different thicknesses were used to simulate restoration. A, 1.5 mm zirconia; B, 1.4 mm zirconia + 0.1 mm OP; C, 2.0 mm zirconia; and D, 1.9 mm zirconia + 0.1 mm OP. OP, opaque porcelain.

A titanium disc (t) and an all-ceramic zirconia disc (c), both with a diameter of 15 mm and thickness of 1 mm, were used to simulate the titanium and all-ceramic abutment backgrounds (Fig 1). Zirconia discs (UPCERA,China,15 mm in diameter,3M2) were precision-cut,polished sequentially with 400–1200 grit SiC abrasives, ultrasonically cleaned (10 min), and dried with oil-free compressed air.Specimens of zirconia discs were categorized into the following four groups based on their thickness and OP application to simulate the implant restoration (Fig 2):

Group A: zirconia with a thickness of 1.5 mm
Group B: zirconia with a thickness of 1.4 mm (above) + OP with a thickness of 0.1 mm (beneath)
Group C: zirconia with a thickness of 2.0 mm
Group D: zirconia with a thickness of 1.9 mm (above) + OP with a thickness of 0.1 mm (beneath)

**Fig 3:**
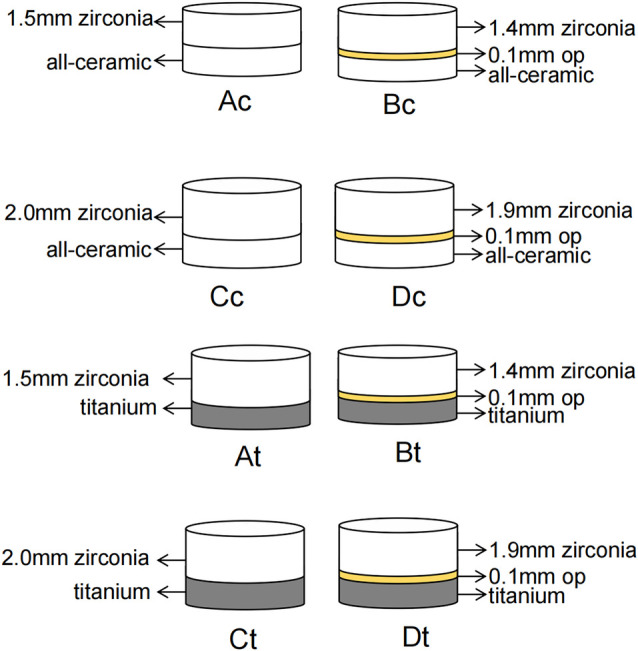
Schematic diagram of color parameter analysis for specimens with different restoration and background combinations. Ac,1.5 mm zirconia with all-ceramic background; Bc, 1.4 mm zirconia+OP with all-ceramic background; Cc, 2.0 mm zirconia with all-ceramic background; Dc, 1.9 mm zirconia+OP with all-ceramic background; At, 1.5 mm zirconia with titanium background; Bt, 1.4 mm zirconia+OP with titanium background; Ct, 2.0 mm zirconia with titanium background; Dt, 1.9 mm + OP with titanium background; z,zirconia;t, titanium;c,all-ceramic;OP,opaque porcelain.

Discs in Groups A and C were composed of pure zirconia, while in Groups B and D, a 0.1 mm OP layer was applied beneath the zirconia via standard stacking and sintering.The thickness of OP was selected per manufacturer specifications to balance masking efficacy with material conservation and avoid excessive thickness that could compromise translucency or mechanical integrity.OP powder was layered on discs of group B and D, and sintered under vacuum following manufacturer’s sintering protocol to ensure interfacial bonding and chromatic stability. In groups A and B, the total thickness was 1.5 mm, while in groups C and D, the total thickness was 2.0 mm. The required sample size for the experiment was calculated using PASS15.0 software (NCSS, USA) based on the results of a pilot experiment. For each group, 10 sets of restoration-background specimens were prepared, and each disc in every group was numbered. The zirconia discs (groups A, B, C, D) were bonded to different backgrounds (titanium or all-ceramic) using removable transparent adhesive. The following combinations were defined based on the permutations of discs and backgrounds: At, 1.5 mm zirconia with titanium background; Ac, 1.5 mm zirconia with all-ceramic background; Bt, 1.4 mm zirconia + 0.1 mm OP with titanium background; Bc, 1.4 mm zirconia + 0.1 mm OP with all-ceramic background; Ct, 2.0 mm zirconia with titanium background; Cc, 2.0 mm zirconia with all-ceramic background; Dt, 1.9 mm + 0.1 mm OP with titanium background; and Dc, 1.9 mm zirconia +0.1 mm OP with all-ceramic background (Fig 3).

**Fig 4:**
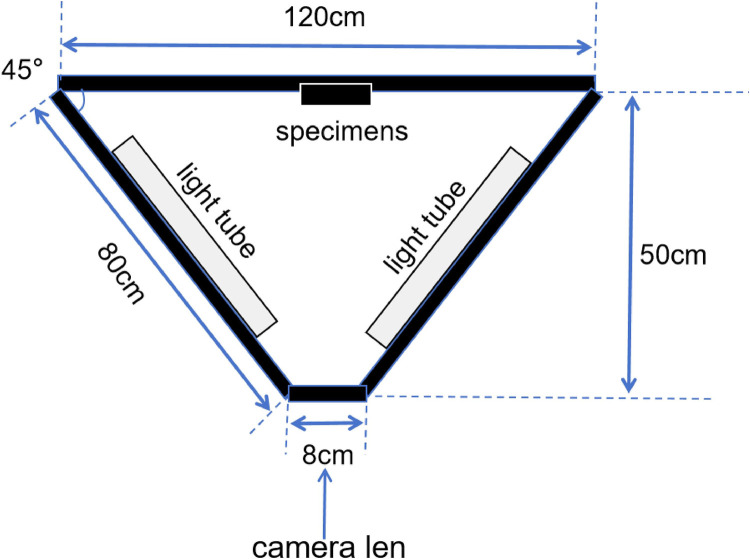
Schematic diagram of photography platform.

**Fig 5:**
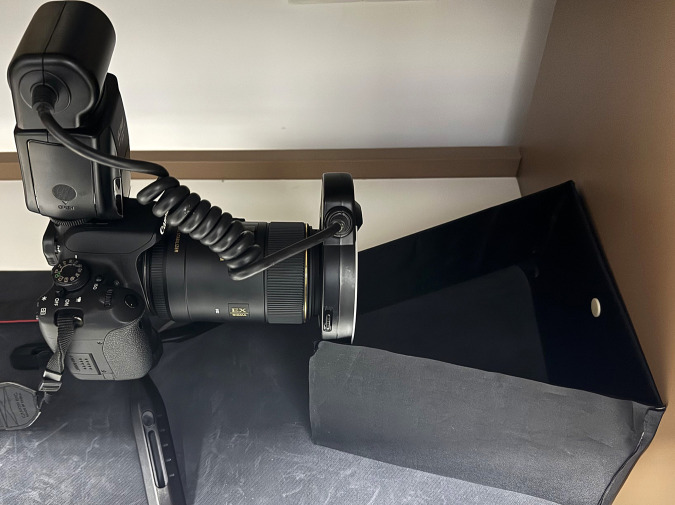
Picture of the photography platform and the camera.

These assembled specimens were placed at the center of a dark backboard in a specialized constructed photography platform. The observation method of d/0° was employed during the photography process [15]. The photography platform was assembled with black polystyrene boards covered with a black flocking cloth as the inner wall. The back wall measured 120 × 20 cm (length × height), the left and right sidewalls measured 80 × 20 cm (length × height), and the angle between them was 45°.Two light tubes were placed on each side wall. The upper wall was covered during image shooting to ensure that external light did not interfere with the photography platform (Figs 4 and 5).

Specimens were photographed using a single-lens reflex camera (Canon, Japan) and a 105 mm lens (SIGMA, Japan). The camera parameters were set as follows: aperture of f/2.8, shutter speed of 1/30 sec, ISO of 100, and photographic ratio of 1:1, with RAW image format [16]. During shooting, the camera used manual focus and time-lapse shutter mode. The camera was firmly fixed on a tripod and entered the shooting darkroom from the front of the photography platform. The shooting distance was 50 cm, and the stability of the observation angle and distance was maintained, ensuring that the measured specimen was positioned at the center of the lens and perpendicular to its long axis.Photographic parameters and illumination conditions were rigorously standardized to minimize bias. Regular calibration checks ensured consistency across sessions.

**Fig 6:**
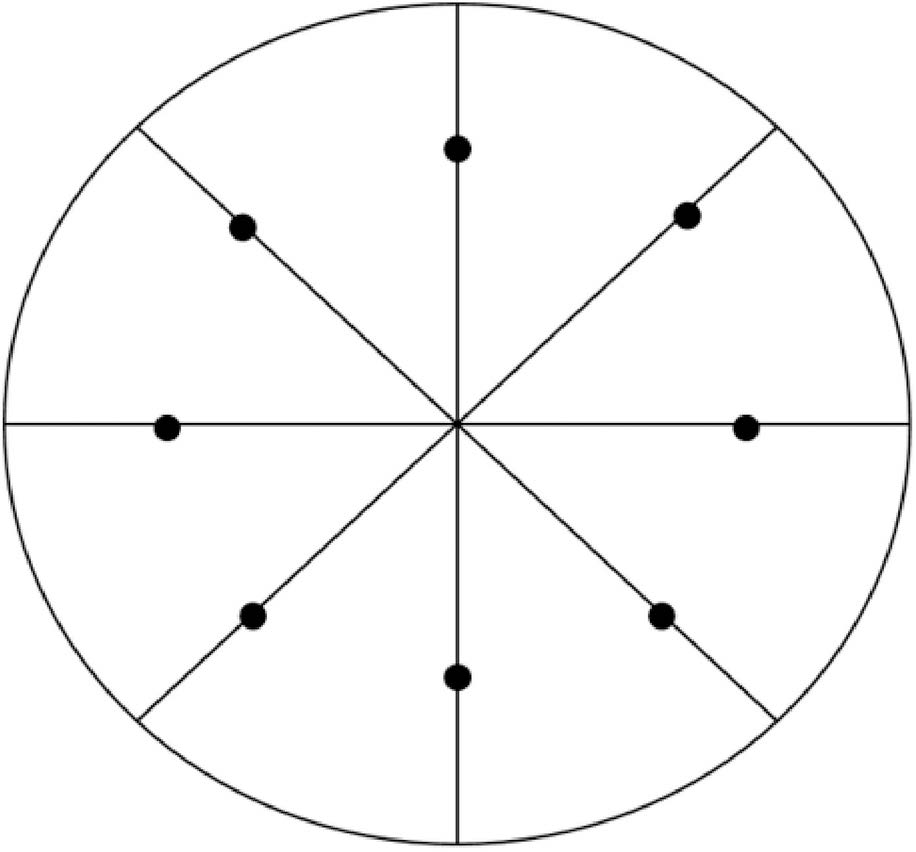
Schematic diagram of the selection of regions of interest on an image of a zirconia disc (black dots denote the selected regions).

A point on the disc’s edge was selected directly from the obtained image and connected to the disc’s center, with the line connecting the two points set in the 0° direction. With this line as the reference, we moved in the clockwise direction; determined the azimuth at positions of 45°, 90°, 135°, 180°, 225°, 270°, 315°, and 360°; and selected a sampling point at 5 mm from the center of the disc (Fig 6). In this way, a total of 8 sampling points were determined on each image of these discs, and each selected region was also numbered. The color sampling tool in the Adobe Photoshop 2024 (Adobe, USA) software was used to collect color parameters in corresponding regions [17]. The color parameters in the regions were provided by the software system and recorded as L*, a*, and b* values.

After measuring the color data in each region from each group, the color differences (△E) between each specimen were calculated according to the corresponding number and selected region. For two regions from specimens with different restoration and background combinations, the color difference was calculated using the following formula: ΔE = [(ΔL*)2 + (Δa*)2 + (Δb*)2]1/2, ΔL* = L1* -L2*, Δa* = a1*-a2*, Δb* = b1*-b2* [18].In this study, we adhered to the American Dental Association’s guidelines by defining ΔE = 2.0 as the threshold value for clinically acceptable color differences [19].

The statistical software, SPSS 27.0 (IBM, USA), was employed to analyze the data, and GraphPad Prism 9 (Graphpad, USA) was used for figure drawing. The mean and standard deviation of the L*, a*, and b* values, as well as the color differences between selected regions from specimens, were calculated and compared pairwise. The Kolmogorov-Smirnov test was used,and verified that the L*, a*, and b* parameters did not fit the normal distribution (p < 0.001); further, the Levene test was used, and confirmed that the variance between, and within groups did not satisfy the homogeneity(p < 0.05). Therefore, the Kruskal-Wallis H test was applied to compare the color parameters between groups, and Welch’s t-test was used to conduct intergroup and intragroup pairwise comparisons of the color parameters on the zirconia and titanium backgrounds. A p value of < 0.05 was considered statistically significant.

## 3. Results

### 3.1 Color parameters of specimens under different backgrounds

Tables 1 and 2 present the color parameters of the different groups of zirconia restoration with all-ceramic (Table 1) and titanium (Table 2) backgrounds, respectively. The Kruskal-Wallis H test showed that the L*, a*, and b* parameters of the zirconia discs in groups A, B, C, and D were significantly different between all-ceramic and titanium backgrounds (p < 0.001).

**Table 1:** Comparison of color parameters of different groups under all-ceramic background.

						95%CI				
Color parameter	Group	No.	Mean	SD	SE	Lower bound	Upper bound	Minimum	Maximum	p
L*	A	80	93.03	0.81	0.09	92.85	93.21	92.00	95.00	<0.001^#^
	B	80	92.37	0.70	0.07	92.21	92.53	91.00	94.00	
	C	80	91.72	0.95	0.06	91.59	91.85	90.00	93.00	
	D	80	91.81	0.58	0.06	91.68	91.93	91.00	93.00	
a*	A	80	0.36	0.48	0.05	0.25	0.47	0.00	1.00	<0.001^#^
	B	80	0.46	0.50	0.05	0.35	0.57	0.00	1.00	
	C	80	0.16	0.37	0.04	0.07	0.24	0.00	1.00	
	D	80	0.15	0.34	0.04	0.07	0.23	0.00	1.00	
b*	A	80	1.25	0.46	0.05	1.14	1.35	0.00	2.00	<0.001^#^
	B	80	1.43	0.49	0.05	1.32	1.54	1.00	2.00	
	C	80	1.11	0.59	0.06	0.98	1.24	0.00	3.00	
	D	80	1.21	0.49	0.05	1.10	1.32	0.00	2.00	

# indicates statistical significance

**Table 2:** Comparison of color parameters of different groups under titanium background.

						95% CI				
Color parameter	Group	No.	Mean	SD	SE	Lower bound	Upper bound	Minimum	Maximum	p
L*	A	80	90.68	0.66	0.07	90.53	90.83	90.00	92.00	<0.001^#^
	B	80	91.68	0.70	0.07	91.53	91.84	90.00	93.00	
	C	80	93.28	0.78	0.05	93.16	93.40	92.00	94.00	
	D	80	92.47	0.77	0.08	92.30	92.64	90.00	94.00	
a*	A	80	0.03	0.19	0.02	−0.01	0.08	0.00	1.00	<0.001^#^
	B	80	0.08	0.28	0.03	0.02	0.15	0.00	1.00	
	C	80	0.03	0.19	0.02	−0.01	0.08	0.00	1.00	
	D	80	0.06	0.24	0.02	0.01	0.11	0.00	1.00	
b*	A	80	0.73	0.44	0.04	0.63	0.83	0.00	1.00	<0.001^#^
	B	80	0.98	0.51	0.05	0.87	1.10	0.00	2.00	
	C	80	0.87	0.33	0.03	0.80	0.94	0.00	1.00	
	D	80	0.96	0.46	0.05	0.86	1.06	0.00	3.00	

# indicates statistical significance.

### 3.2 Color parameters of specimens with different thicknesses

#### 3.2.1 For groups with a 1.5 mm thickness.

On considering different backgrounds, in group A, the L*, a*, and b* differences between all-ceramic and titanium backgrounds were significant (p < 0.001), while in group B, the L*, a*, and b* differences under both backgrounds were significant (p < 0.001).

#### 3.2.2 Between groups with or without OP.

Under all-ceramic backgrounds, L* was significantly higher in group A than in group B (P < 0.001), b* was significantly lower in group A than in group B(P = 0.015), while a* was not significantly different between groups A and B.Under titanium background, L*, a* and b* were significantly different between groups A and B (p < 0.001) (Table 3, Fig 7).

**Table 3:** Color parameters of 1.5 mm zirconia restoration under all-ceramic and titanium backgrounds (x ± s).

1.5 mm Groups	No.	L*			a*			b*		
		c	t	p	c	t	p	c	t	p
A	80	93.03 ± 0.81	90.68 ± 0.66	<0.001^#^	0.36 ± 0.48	0.03 ± 0.19	<0.001^#^	1.25 ± 0.46	0.73 ± 0.44	<0.001^#^
B	80	92.37 ± 0.70	91.68 ± 0.70	<0.001^#^	0.46 ± 0.50	0.08 ± 0.28	<0.001^#^	1.43 ± 0.49	0.98 ± 0.51	<0.001^#^
p		<0.001^#^	<0.001^#^		0.201	0.194		0.015^#^	<0.001^#^	

# indicates statistical significance; c, all-ceramic background; t, titanium background

**Fig 7:**
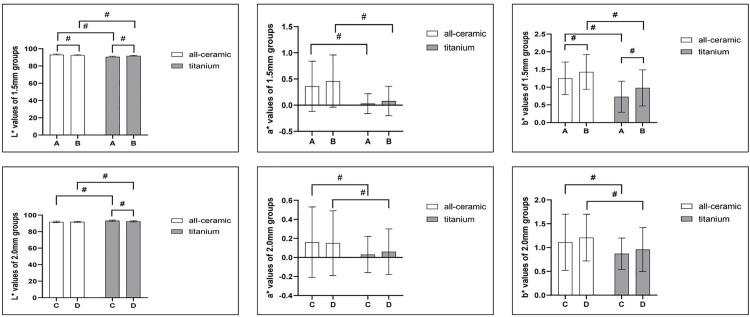
The Mean and SD of color parameters L*, a*, and b* values between different restoration groups under all-ceramic and titanium backgrounds. Figure 7.1-7.3, the L*,a*,b* values in A and B groups(1.5 mm groups), and Figure 7.4-7.6, the L*,a*,b* values in C and D groups, respectively,(2.0 mm groups). #, indicates statistical significance.

#### 3.2.3 For groups with a 2.0 mm thickness.

Under different backgrounds, in group C, the L* (p < 0.001), a* (p = 0.008), and b* (p < 0.001) differences were significant between all-ceramic and titanium backgrounds. In group D, L* (p < 0.001) and b* (p < 0.001) were significantly different between all-ceramic and titanium backgrounds.

#### 3.2.4 Between groups with or without OP.

Under all-ceramic backgrounds, L*, a*, and b* were not significantly different between groups C and D. Under titanium backgrounds, only the L* value was significantly different between groups C and D (p < 0.001) (Table 4, Fig 7).

**Table 4:** Color parameters of the 2.0 mm zirconia restoration group under all-ceramic and titanium backgrounds (x ± s).

2.0 mm Groups	No.	L*			a*			b*		
		c	t	p	c	t	p	c	t	p
C	80	91.72 ± 0.95	93.28 ± 0.78	<0.001^#^	0.16 ± 0.37	0.03 ± 0.19	0.008^#^	1.11 ± 0.59	0.87 ± 0.33	<0.001^#^
D	80	91.81 ± 0.58	92.47 ± 0.77	<0.001^#^	0.15 ± 0.34	0.06 ± 0.24	0.073	1.21 ± 0.49	0.96 ± 0.46	<0.001^#^
p		0.337	<0.001^#^		0.829	0.471		0.250	0.172	

#, indicates statistical significance; c, all-ceramic background; t, titanium background.

The results showed that the metallic background of the titanium abutment affected the lightness and color of the implant restoration, and the use of OP affected the lightness of the restoration.

### 3.3 Rescue effect of OP on color parameters of implant restoration

#### 3.3.1 For groups with a 1.5 mm thickness.

In group A, the color difference, △E (Ac/At), between the all-ceramic and titanium backgrounds was 3.22 ± 1.30, which exceeded the clinically acceptable range. In group B, the color difference, △E (Bc/Bt), between the all-ceramic and titanium backgrounds was 1.55 ± 0.66, which was within the clinically acceptable range.

Under a titanium background, the color difference, △E (At/Bt), between groups A and B was 2.44 ± 0.99, which is greater than the clinically acceptable range. Under an all-ceramic background, the color difference, △E (Ac/Bc), between groups A and B was 1.47 ± 0.73, which is less than the clinically acceptable range.

#### 3.3.2 For groups with a 2.0 mm thickness.

In group C, the color difference, △E(Cc/Ct), between the all-ceramic and titanium backgrounds was 2.87 ± 1.90, which exceeded the clinical acceptable range. In group D, the color difference, △E(Dc/Dt), between the all-ceramic and titanium backgrounds was 1.45 ± 0.87, which was within the clinical acceptable range.

In groups C and D, the color difference, △E(Ct/Dt), was 2.12 ± 1.27, which exceeded clinical limits. However, the color difference, △E(Cc/Dc), between groups C and D under an all-ceramic background was 1.06 ± 0.58, which was within the clinical acceptable range.

#### 3.3.3 Color-rescuing effect of OP under different backgrounds.

The color difference, △E (Ac/Bt), between group A under an all-ceramic background and group B under a titanium background was 1.99 ± 0.86, and the color difference, △E (Cc/Dt), between group C under an all-ceramic background and group D under a titanium background was 1.31 ± 0.83, both of which were lower than the clinically acceptable range (Table 5).

**Table 5:** Color difference (x ± s) among different zirconia groups under all-ceramic or titanium backgrounds.

1.5 mm Groups	No.	Mean △E	No. of △E ≥ 2	No. of △E < 2	χ²	P
Ac vs At	80	3.22 ± 1.30^ɵ^	66	14	70.943	<0.001^#^
Bc vs Bt	80	1.55 ± 0.66	23	57		
At vs Bt	80	2.44 ± 0.99^ɵ^	37	43		
Ac vs Bt	80	1.99 ± 0.86	40	40		
Ac vs Bc	80	1.47 ± 0.73	22	58		
At vs Bc	80	2.13 ± 0.83^ɵ^	51	29		
**2.0 mm Groups**	**No.**	**△E**	**No. of △E ≥ 2**	**No. Of △E < 2**	χ²	**P**
Cc Vs Ct	80	2.87 ± 1.90^ɵ^	46	34	70.124	<0.001^#^
Dc Vs Dt	80	1.45 ± 0.87	11	69		
Ct Vs Dt	80	2.12 ± 1.27^ɵ^	22	58		
Cc Vs Dt	80	1.31 ± 0.83	25	55		
Cc Vs Dc	80	1.06 ± 0.58	5	75		
Ct Vs Dc	80	1.70 ± 0.74	37	43		

#, indicates statistical significance; ɵ, indicates exceeds clinical acceptable range; c, all-ceramic background; t, titanium background.

The number of compared pairs with △E values ≥2 and <2 is summarized in Table 5, and the chi-square test revealed that their proportions were significantly different, including in both the 1.5 mm and 2.0 mm groups.

The results showed that the titanium abutments significantly affected zirconia restoration color (p < 0.001). However, OP application reduced this difference, bringing ΔE within clinically acceptable limits.

## 4. Discussion

Titanium abutments are widely used in implant restorations because of their favorable biological and mechanical properties. However, their clinical use can lead to an undesirable grayish-black metallic appearance through restoration [18,20,21]. This study aimed to examine the effects of titanium abutments on the color of implant restorations and to assess the potential color-rescuing efficacy of OP, thereby providing a scientific basis for clinical practice.

The CIELAB (1976) colorimetric system, developed by the International Commission on Illumination, is one of the most widely used method for object hue measurement.The CIELAB color system quantifies color using three parameters: L* represents lightness (0 = black, 100 = white), reflecting the brightness of the restoration; a* indicates chromaticity along the green-red axis, signifying shifts toward cool or warm tones; b* indicates chromaticity along the blue-yellow axis, signifying dominance of yellow or blue. In this study, applying OP significantly altered these parameters. Specifically, restorations exhibited higher L* values with OP, enhancing overall brightness. Additionally, chromaticity shifts along both the a* and b* axes were reduced, with restorations showing lower a* (green) and b* (blue) values. Without OP, the titanium abutment resulted in lower L* values (indicating a darker appearance) and undesirable chromaticity shifts (green or blue).

Color difference (ΔE) refers to the degree of dissimilarity between two colors when perceived visually or measured quantitatively, representing the distance between the points representing these colors in a color space with rectangular coordinates [12]. The American Dental Association recommends a threshold of △E = 2 for detectable color differences in clinical dental restorations [19]. The greater the ΔE value, the larger the color difference. The CIELAB colorimetric system provides a practical and reliable method for accurately evaluating color differences, thereby enabling objective and effective assessment of whether the colors of the experimental zirconia ceramic groups reached clinically acceptable similarity.

Implant-supported restorations differ from traditional dental fixed restorations, as the implant abutment provides support instead of the natural tooth. In traditional prosthodontic restorations, the color of the tooth abutment is more consistent with that of the surrounding teeth with no metallic background color. The abutments used in implant restorations are typically composed of titanium and titanium alloys, which exhibit considerable color disparity with natural teeth. Once implant restoration is accomplished, the overall color of the implant restoration may become less natural, and color coordination with adjacent teeth may be suboptimal.

Zirconia, as an all-ceramic material, has light transmission properties and a smaller color difference when compared with natural teeth [22–25]. In the absence of OP, the ΔE values of zirconia discs under titanium and all-ceramic substrates exceed the clinically acceptable range no matter 1.5 mm or 2.0 mm thickness [26]. This phenomenon may attributed to the translucency of zirconia, which renders the final color of zirconia restorations more susceptible to the influence of the underlying color of the background [27]. Previous studies have also indicated that the aesthetic effect of restorations employing titanium abutments is inferior to that of restorations using all-ceramic (zirconia) abutments, which is primarily attributed to the metallic color of the implant abutments and non-transmittance of metal abutments [20,28].

In dental laboratories, OP plays a crucial role in the fabrication of high-quality dental restorations. Although the improvement of dental materials has continuously enhanced the behavior and performance of dental ceramics over the past 50 years, the main function that OP needs to fulfill, namely masking the metal substrate, has remained unchanged. This specialized type of porcelain was designed to make the final restoration appear natural and aesthetically pleasing. In this research, after applying OP, the ΔE between the metallic and all-ceramic backgrounds was within the clinically acceptable range. Furthermore, when comparing restoration with OP under a titanium background and restoration without OP under an all-ceramic background, the ΔE was within the clinically acceptable range, which confirmed the color-rescuing effect of OP. In the field of implant restoration, Arif et al. [29] found that the use of OP on a titanium background resulted in a small color difference from its use on a zirconia background in several crown systems, demonstrating that OP can be used to prevent the unfavorable metal show-through.Similarly, In the fabrication of PFM restorations, applying OP can also effectively mask the influence of the metal’s color and enhance the restoration’s brightness and chroma [12]. These findings suggest that OP should be routinely considered in implant restorations, particularly in cases where a metal abutment is used. Therefore, OP remains an indispensable material in implant prosthesis fabrication, as it can provide aesthetic benefits in terms of creating natural-looking dental restorations.

The result of this study confirms that OP application significantly reduces titanium-induced color interference in all-ceramic restorations (ΔE < 2.0), aligning with recent studies of material opacity and thickness in color masking. For example, Gouveia et al. employed Kubelka-Munk (K-M) reflectance theory to predict composite resin thickness effects on color matching over opaque PEEK substrates [30]. And da Silva et al. demonstrated that high-opacity resin cements reduce ceramic sensitivity to substrate saturation [31]. These findings parallel OP’s role here: by increasing material opacity, OP neutralizes titanium’s grayish-black metallic appearance, thereby reducing color difference.

The thickness of the ceramic layer is also an essential factor that influences the ultimate aesthetic outcome of the restoration [32–35]. This study discovered that group C, with a total thickness of 2.0 mm, exhibited a lower ΔE value (2.87 ± 1.90) between the two backgrounds than group A (3.22 ± 1.30), with a total thickness of 1.5 mm. The result demonstrated that increasing the thickness of the ceramic can reduce the color disparity. In traditional prosthodontic restorations, studies have demonstrated that the thickness of the restoration influences its color. Specifically, greater preparation depth of the abutment tooth results in thicker restoration, thereby reducing the color difference between the restoration and natural tooth [36–38]. While for implant restoration, Jirajariyawong et al. [26] demonstrated that increasing the ceramic restoration thickness over the metal substrate decreased the color mismatch, and the color differences between the titanium and all-ceramic backgrounds decreased with increasing thickness of the restoration. In this study, the ∆E values between the 1.5 mm groups was smaller than that between the corresponding 2.0 mm groups, which indicated that increasing the ceramic thickness improved the masking effect on the metal substrate.

A key constraint of this study is the exclusive use of a single zirconia brand to standardize ceramic composition and mitigate inter-manufacturer variations, which may constrain the generalizability of the findings. And as this study was preliminary, *in vivo* clinical aesthetic experiments were not conducted at this time. Future work will give priority to conducting subsequent clinically-oriented research and assesses the long-term stability of OP in a dynamic oral environment.

Notwithstanding these limitations, based on the outcomes of this study, the null hypothesis T_0_ was confirmed, suggesting that the application of OP can partially mitigate the color alteration of implant restorations resulting from metal abutments and that the thicker the ceramic layer of the zirconia restoration, the smaller the color disparity.

## 5. Conclusion

The results of this study revealed that titanium abutments significantly alter the final color of zirconia restorations. However, OP effectively mitigates this effect, with greater improvement at increased restoration thickness. These findings provide a reference for clinicians to optimize implant restoration aesthetics by incorporating OP and controlling restoration thickness.

## Supporting information

S1 Data Color parameters of zirconia restoration groups under different backgrounds. c, all-ceramic background; t, titanium background. (XLSX)

## Abbreviations

Ac: 1.5 mm Zirconia with All-Ceramic Background
At: 1.5 mm Zirconia with Titanium Background
Bc: 1.4 mm Zirconia + 0.1 mm OP with All-Ceramic Background
Bt: 1.4 mm Zirconia + 0.1 mm OP with Titanium Background
Cc: 2.0 mm Zirconia with All-Ceramic Background
Ct: 2.0 mm Zirconia with Titanium Background
CIE: International Commission on Illumination
ΔE: Color Difference
Dc: 1.9 mm Zirconia + 0.1 mm OP with All-Ceramic Background
Dt: 1.9 mm Zirconia + 0.1 mm OP with Titanium Background
OP: Opaque Porcelain
PFM: Porcelain-Fused-to-Metal
ROI: Regions of Interest.
